# Prevalence and Predictive Value of Dyspnea Ratings in Hospitalized Patients: Pilot Studies

**DOI:** 10.1371/journal.pone.0152601

**Published:** 2016-04-12

**Authors:** Jennifer P. Stevens, Kathy Baker, Michael D. Howell, Robert B. Banzett

**Affiliations:** 1 Center for Healthcare Delivery Science, Beth Israel Deaconess Medical Center, Boston, Massachusetts, United States of America; 2 Department of Medicine, Division for Pulmonary, Critical Care, and Sleep Medicine, Beth Israel Deaconess Medical Center, Boston, Massachusetts, United States of America; 3 Harvard Medical School, Boston, Massachusetts, United States of America; 4 Department of Nursing, Beth Israel Deaconess Medical Center, Boston, Massachusetts, United States of America; 5 Department of Medicine, Section of Pulmonary and Critical Care, University of Chicago, Chicago, Illinois, United States of America; 6 Center for Healthcare Delivery Science and Innovation, University of Chicago, Chicago, Illinois, United States of America; University of Rochester, UNITED STATES

## Abstract

**Background:**

Dyspnea (breathing discomfort) can be as powerfully aversive as pain, yet is not routinely assessed and documented in the clinical environment. Routine identification and documentation of dyspnea is the first step to improved symptom management and it may also identify patients at risk of negative clinical outcomes.

**Objective:**

To estimate the prevalence of dyspnea and of dyspnea-associated risk among hospitalized patients.

**Design:**

Two pilot prospective cohort studies.

**Setting:**

Single academic medical center.

**Patients:**

Consecutive patients admitted to four inpatient units: cardiology, hematology/oncology, medicine, and bariatric surgery.

**Measurements:**

In Study 1, nurses documented current and recent patient-reported dyspnea at the time of the Initial Patient Assessment in 581 inpatients. In Study 2, nurses documented current dyspnea at least once every nursing shift in 367 patients. We describe the prevalence of burdensome dyspnea, and compare it to pain. We also compared dyspnea ratings with a composite of adverse outcomes: 1) receipt of care from the hospital’s rapid response system, 2) transfer to the intensive care unit, or 3) death in hospital. We defined burdensome dyspnea as a rating of 4 or more on a 10-point scale.

**Results:**

Prevalence of burdensome current dyspnea upon admission (Study 1) was 13% (77 of 581, 95% CI 11%-16%). Prevalence of burdensome dyspnea at some time during the hospitalization (Study 2) was 16% (57 of 367, 95% CI 12%-20%). Dyspnea was associated with higher odds of a negative outcome.

**Conclusions:**

In two pilot studies, we identified a significant symptom burden of dyspnea in hospitalized patients. Patients reporting dyspnea may benefit from a more careful focus on symptom management and may represent a population at greater risk for negative outcomes.

## Introduction

Dyspnea–a subjective experience of breathing discomfort[1]–causes significant and memorable fear and anxiety among patients.[2–4] Patients’ comments about the experience of severe dyspnea are illuminating, and may be associated with a sense of impending doom or death. [4] We are attuned to dyspnea perhaps because it also functions as a critically important warning system for many different organ systems in peril in the body. Dyspnea is experienced by patients with difficulties with oxygen transport (*e*.*g*. heart failure, emphysema, pulmonary embolism, and anemia), with metabolic dysfunction (*e*.*g*. lactic acidosis), high pulmonary vascular pressures (e.g., congestive heart failure), and increased work of breathing (e.g., increased airway resistance and hyperinflation from asthma and COPD). Given the prevalence of many medical conditions that cause dyspnea, the burden for the hospitalized patient of this powerfully aversive symptom may be significant. Previous authors have reported that about half of the sickest patients experience significant dyspnea [5,6], but the true prevalence and intensity of dyspnea in the broader hospitalized population is unknown.

Given the severity of the emotional response to breathing discomfort, dyspnea, like pain, ought to be assessed, documented, and managed [7]. Most people can provide reliable quantitative reports of internal sensations and symptoms, as has been demonstrated with evaluation and management of pain. [8–11] As with pain, the first response to dyspnea is to address the underlying condition, but direct symptom management is possible and often necessary. However, designing patient-centered approaches to identify and respond to dyspnea necessitate a true understanding of the burden of this symptom among hospitalized patients.[2,3]

Further, dyspnea has the potential to serve as a predictor of adverse clinical outcome. Several studies have shown dyspnea to be a good predictor of morbidity and mortality in specific populations, often serving as a better predictor than items usually considered 'gold standards' for assessing severity of specific diseases.[12–19] For example, dyspnea outperformed FEV_1_ as a predictor of 5-year mortality in chronic obstructive pulmonary disease (COPD) patients [16] and outperformed angina during exercise stress testing in predicting cardiac death[20]. Dyspnea is also an important warning sign for patients with non-cardiopulmonary processes. While nausea, pain, loss of appetite and fatigue are hallmark symptoms of many types of GI disease, dyspnea was a stronger predictor of mortality than GI symptoms in esophageal and gastric cancer patients in two separate studies.[21,22] Dyspnea, included as part of a large, multicomponent score, predicted hospitalization of older primary care patients.[23] In addition to patients carrying diagnoses associated with dyspnea, all hospitalized patients are at risk of events likely to cause dyspnea, such as hospital acquired pneumonia, pulmonary emboli, and myocardial infarction following surgery or prolonged immobilization. We therefore hypothesized that patients’ reported dyspnea ratings would serve as an easily obtained warning of clinical decline in a wide spectrum of hospitalized patients.

We undertook two pilot studies in which dyspnea ratings were obtained from patients by nursing staff in medical-surgical units. These studies provide the first estimates of prevalence of dyspnea and of association of dyspnea with in-hospital risk in a broad-spectrum sample of hospitalized patients; they also provide information on implementation of routine dyspnea assessment among inpatients, and guidance for the design of future studies, particularly to clarify whether dyspnea may be used to predict impending patient clinical deterioration.

## Methods

These studies, based on clinical data, were approved by the Institutional Review Board of Beth Israel Deaconess Medical Center, Boston, Massachusetts, and a waiver of informed consent was granted.

### Data Collection, Study Populations, and Patient Characteristics

These two pilot studies were conducted approximately one year apart, and included inpatients admitted to four units in our institution: cardiology, hematology/oncology, general medicine, and bariatric surgery. The four units were selected based on interest among unit nursing leaders and on patient populations that represented a spectrum of hospital admissions. Before the first study, nurses on these units received training about dyspnea.[24]

#### Study 1 –Assessment of Dyspnea on Admission (N = 581)

As part of routine care, nurses at our institution perform an initial assessment of the patient’s functional status, burden of disease, baseline symptoms and signs, and cognitive and mental status during the first nursing shift following admission to the hospital. This is documented electronically. During a six-week pilot period between February and March 2012, nurses on the four inpatient units were asked to also complete a paper-based dyspnea assessment in addition to their routine initial assessment. This dyspnea assessment had two components: (1) an evaluation of the patient’s current “breathing discomfort” on a 0 to 10 scale with the patient at rest and (2) an evaluation of activity-related “shortness of breath” in the past day using the Medical Research Council (MRC) Breathlessness scale. [25] The MRC asks patients to identify which of five grades of activity causes shortness of breath, ranging from strenuous exercise to undressing. We extended this scale (eMRC) to include eating or talking (grade 6) and rest (grade 7). The procedures used are described elsewhere. [24]

Of the 1,028 consecutive patients admitted to the four units, 595 had a completed current dyspnea assessment, a compliance rate comparable to published pain assessment studies [26]. Of these patients, 581 patients were admitted as inpatients (14 patients admitted with observation status were excluded, as outcome data were not available). 500 of the 581 inpatients also provided data about recent exertional dyspnea.

#### Study 2 –Assessment of Dyspnea Each Nursing Shift (N = 367)

In April 2013, nurses at our institution began assessing dyspnea on all inpatients at least once per nursing shift. This was documented as a bundled assessment (pain, dyspnea, agitation/sedation, and fall risk) in the hand-written vital signs flowsheet for each patient. For dyspnea, patients were asked to rate their current “breathing discomfort” on the same 0 to 10 scale used in Study 1. We extracted data for the four-week period following the start of these new assessments. During this period, 367 inpatients were admitted to the four units included in our study (an additional 121 patients admitted for observation were excluded). Study staff transcribed dyspnea measurements, concurrent pain assessments, and the associated vital signs for all flowsheets scanned and available for analysis for 2,440 patient-days. Data were missing for two primary reasons: 1) flowsheets were missing from the scanned record or data had been recorded on a prior version of the flowsheet form that had no space for dyspnea ratings (82 patient-days missing); or 2) dyspnea measurements were not recorded by the nurses for at least one day on 153 patients.

### Dyspnea Prevalence Measures

We considered a patient’s breathing discomfort rating of 4 or greater to be “burdensome dyspnea”. This *a priori* cut-off was based on prior proposals from the palliative care literature that a rating of ≤ 3/10 is a benchmark for successful dyspnea or pain treatment.[27] For recent activity-related dyspnea, we grouped grades 1&2 (vigorous exercise), grades 3&4 (light exercise), and grades 5–7 (at rest or performing minimal activities of daily living (ADL)).

For both populations, we extracted patient demographics, comorbidities defined using Elixhauser’s method [28], and severity of illness defined using LAPS2[29] from the electronic health record of participants.

### Adverse Outcome Measures

The primary outcome in both cohorts was a composite outcome representing major clinical decompensation, defined as 1) receiving care from the hospital’s rapid response system, 2) being transferred to the ICU, or 3) dying in the hospital. This was treated as a binary variable: *i*.*e*., adverse event or no adverse event. Individuals who had more than one event (*e*.*g*., both the activation of the rapid response system and a transfer to the intensive care unit) were thus counted as having only one adverse outcome. Because there were relatively few adverse outcomes, we grouped dyspnea ratings for analysis using the cutoff point for burdensome dyspnea. Our institution’s rapid response system has been described elsewhere.[30] Explicit parameters are established for mandatory activation of the emergency response using physiological variables or nursing concern. Dyspnea was not one of the parameters currently used to activate rapid response.[30,31]

Secondary descriptive outcomes evaluated included total length of stay, intensive-care-unit length of stay, 30-day readmission to same hospital, and total hospital charges.

#### Statistical Analysis of Association with Adverse Outcome

Our primary unit of analysis was the hospital admission. We report prevalence of burdensome dyspnea (ratings of 4 of 10 or greater). Unadjusted associations of patient characteristics and clinical variables were examined in both studies using Student’s t-test, chi-squared, or Fisher’s exact tests, as appropriate.

To estimate the predictive value of dyspnea at the start of the hospitalization or during hospitalization, we estimated the odds of any adverse event when associated with a dyspnea rating ≥ 4.

All analyses were conducted using SAS (version 9.3, Cary, NC).

## Results

### Dyspnea Prevalence

**Fig 1:**
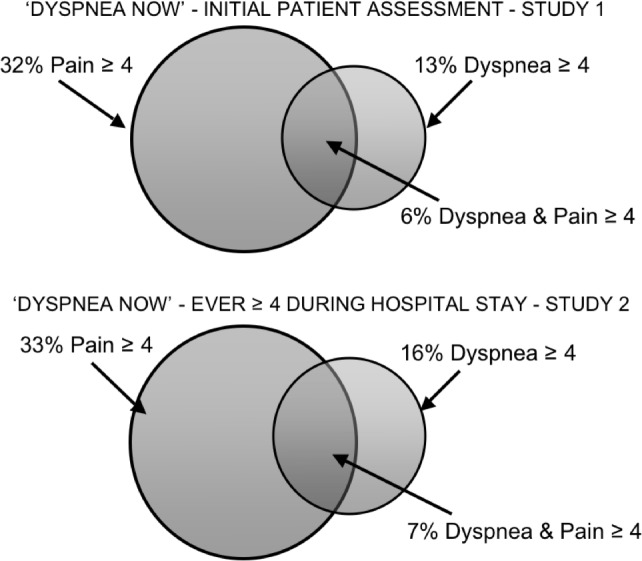
Prevalence of patients experiencing burdensome dyspnea and pain at the time of initial patient assessment (Study 1) and at any time during the hospital stay (Study 2).

In Study 1, 13% (77/581, 95% CI 11%-16%) of patients reported burdensome levels of current dyspnea at the time of initial nursing assessment. This compares to 32% reporting pain, with 6% of the patients suffering both pain and dyspnea at burdensome levels. See Fig 1 upper panel.

Among the 500 patients who also completed the eMRC scale in Study 1, nearly half experienced dyspnea with ordinary activities in the past day: 116 (23.8%) were short of breath when walking on the level at their own pace and 111 (22.8%) were short of breath during minimal activities of daily living (ADL).

In Study 2, 16% (57/367, 95% CI 12%-20%) of patients rated dyspnea as 4 or greater at some point in their hospitalization. This compares to 33% reporting pain, with 7% of the patients suffering both pain and dyspnea at burdensome levels. See Fig 1 lower panel. This indicates that dyspnea increased during hospitalization in many patients, because only 3% (12/367, 95% CI 1–5%) of patients in this study reported burdensome dyspnea at the time of the first shift assessment. Some patients in Study 2 experienced persistent dyspnea while hospitalized: 17 rated dyspnea ≥ 4 on two consecutive shifts and 9 rated dyspnea ≥ 4 on three consecutive shifts. Table 1 identifies clinical and demographic characteristics associated with higher dyspnea ratings.

**Table 1:** Univariate associations with first dyspnea score (both cohorts) and worst dyspnea score (second cohort), dichotomized into <4 and > = 4. Confidence intervals calculated using Wald confidence limits for binomial proportions.

	Pilot Study 1—Initial Patient Assessment			Pilot Study 2 –Every Shift Assessment					
	Current dyspnea, first shift (95% CI)		p-value	Worst dyspnea, any shift (95% CI)		p-value	Current dyspnea, first shift (95% CI)		p-value
	<4	> = 4		<4	> = 4		<4	> = 4	
N	504	77		310	57		353	12	
**Combined outcome (%)**	**11% (8–13%)**	**17% (9–25%)**	**0.1^**	**15% (11–19%)**	**33% (21–46%)**	**0.0008^**	**17% (14–21%)**	**25% (1–50%)**	0.45*
% female	46 (42–51)	53 (42–64)	0.26^	47 (42–53)	56 (43–69)	0.23^	48 (43–54)	66 (40–93)	0.21^
mean age	62 (60–63)	63 (59–66)	0.55**	62(60–64)	68 (64–71)	0.017**	63 (61–65)	63 (52–74)	0.99**
% nonwhite	23 (19–26)	35 (24–46)	0.02^	44 (39–50)	44 (31–57)	0.97^	44 (39–49)	50 (22–78)	0.68^
CHF	16 (13–19)	34 (23–44)	0.002^	17 (13–21)	40 (28–53)	<0.0001	20 (16–25)	33 (7–60)	0.28*
Valve disease	6 (4–8)	10 (4–17)	0.19^	7 (4–10)	2 (0–5)	0.22*	6 (3–8)	8 (0–24)	0.53*
Pulmonary circulation	5 (3–6)	12 (5–19)	0.03*	4 (2–6)	16 (6–25)	0.003*	5 (3–8)	25 (1–50)	0.03*
Paralysis	<1 (0–2)	4 (0–8)	0.08*	1 (0–3)	2 (0–9)	0.58*	1 (0–2)	8 (0–24)	0.15*
Other neurologic disease	5 (3–7)	8 (2–14)	0.27^	7 (4–10)	13 (4–21)	0.19*	8 (5–10)	17 (0–38)	0.25*
COPD	14 (11–18)	29 (18–39)	0.002^	14 (10–18)	30 (18–42)	0.004*	16 (12–20)	33 (7–60)	0.12*
DM	23 (20–27)	18 (10–27)	0.31^	18 (14–22)	21 (10–32)	0.54^	18 (14–22)	17 (0–38)	1*
DM with compli-cations	5 (3–7)	10 (4–17)	0.06*	7 (4–10)	5 (0–11)	1*	7 (4–9)	0 (0–0)	1*
Hypo-thyroidism	12 (9–14)	14 (6–22)	0.48^	11 (8–15)	9 (1–16)	0.6^	11 (8–14)	8 (0–24)	1*
Renal failure	15 (12–18)	29 (18–39)	0.003^	14 (10–18)	26 (15–38)	0.02*	16 (12–20)	17 (0–38)	1*
Obesity	7 (5–9)	5 (0–10)	0.53^	12 (9–16)	9 (1–16)	0.47	12 (9–16)	0 (0–0)	0.37*
Chronic blood loss	<1 (0–1)	3 (0–6)	0.05*	2 (0–3)	0 (0–0)	1*	1 (0–3)	0 (0–0)	1*
Alcohol abuse	3 (2–5)	8 (2–14)	0.06*	4 (2–6)	4 (0–8)	1*	4 (2–6)	8 (0–24)	0.40*
Drug abuse	2 (1–3)	3 (0–6)	0.66*	1 (0–3)	9 (1–16)	0.006*	2 (1–4)	17 (0–38)	0.04*
Psychotic illness	3 (1–4)	4 (0–8)	0.72*	3 (1–5)	2 (0–5)	1*	3 (1–5)	0 (0–0)	1*
Depression	16 (13–19)	14 (6–22)	0.75^	15(11–19)	12 (4–21)	0.69*	15 (11–19)	8 (0–24)	1*
Chronic hyper-tension	61 (56–65)	58 (47–69)	0.73^	59 (53–64)	61 (49–74)	0.68^	58 (53–64)	75 (51–100)	0.25^
% emergent	88 (85–91)	100 (100–100)	0.001^	89 (86–93)	96 (92–100)	0.09^	90 (87–93)	100	0.61*
LAPS2 score	31 (29–34)	41 (35–47)	0.002**	30 (27–32)	42 (36–48)	0.0003**	31 (29–33)	47 (34–59)	0.03**
LOS	3.3 (3.0–3.6)	4.5 (3.0–6.2)	0.13**	4.6 (4.2–5.1)	8.1 (6.2–9.9)	<0.0005**	5.2 (4.7–5.6)	5.3 (2.1–8.4)	0.94**
ICU LOS	<0.2 (0.05–0.26)	<0.2 (0–0.85)	0.33**	0.23 (.13-.33)	0.82 (.17–1.5)	0.08**	<0.5 (.19-.47)	0 (0–0)	<0.0001**
Total charges ($)	23,633 (21,318–25,949)	26,658 (16,241–37,076)	0.57**	26,015 (22,764–29,266)	43,586 (30,744–56,428)	0.01**	29,075 (25,549–32602)	19,451 (7,844–31,059)	0.33**
% readmitted at 30 days	6 (7–12)	9 (3–16)	1^	28 (23–33)	30 (18–42)	0.8^	30 (23–33)	42 (14–70)	0.33*

P-values marked with * indicate differences evaluated using Fisher's Exact test, ^ indicate chi-sq. tests, and ** indicate Student’s t-test

**Fig 2:**
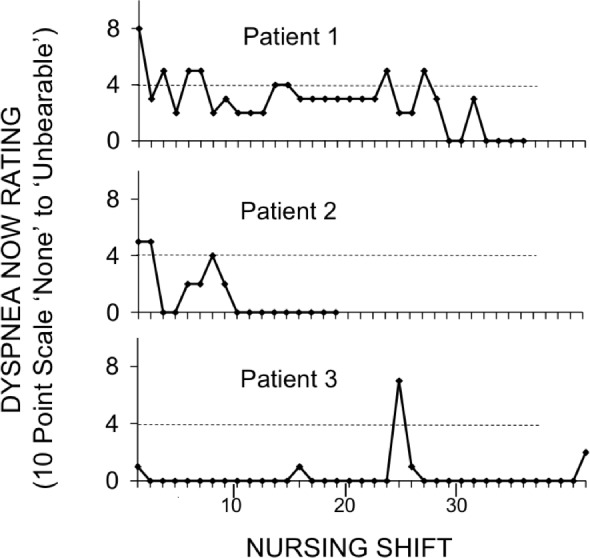
Three example patterns of dyspnea recorded once per nursing shift. Patient 1 presented with a dyspnea score of 8, Patient 2 with a score of 5 and Patient 3 with a score of 1. All of these patients had a length of stay more than 8 days, longer than average. (Study 2)

Patients exhibited a range of time courses of reported dyspnea. Three example patterns are presented in Fig 2. Patient 1 reports a significant amount of dyspnea on arrival and continues to intermittently report dyspnea throughout his/her stay; Patient 2 arrives at the hospital with moderate dyspnea, which improves halfway through his/her hospitalization; and Patient 3 has a single episode of significant dyspnea much later in the hospitalization. Each of these three patients had a length of stay more than 8 days, but each has different dyspnea at the outset and different patterns of dyspnea throughout their stays.

### Association of Dyspnea with Adverse Outcomes

#### Study 1—Outcomes associated with Dyspnea on Admission

Of the 581 inpatients included in Study 1, 9 (1.6%) died, 26 (4.5%) were transferred to the intensive care unit, and 53 (9.1%) required activation of the medical emergency response team (Table 1).

**Fig 3:**
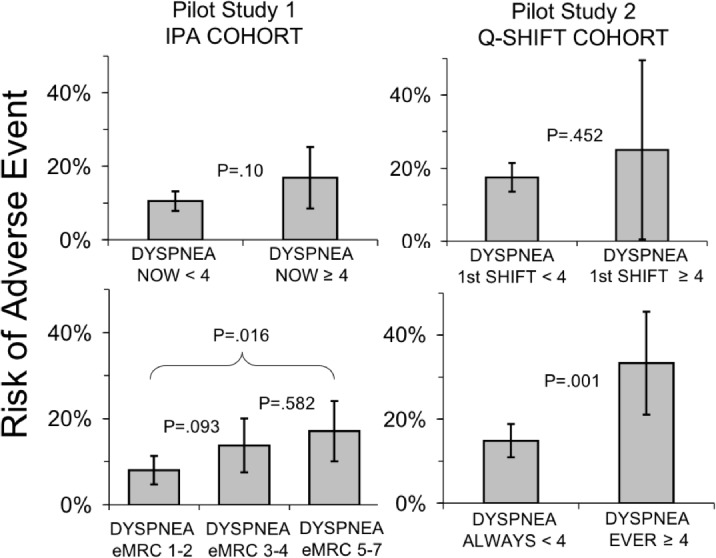
Univariable comparisons of risk of combined negative outcome related to measurements of dyspnea. Panel A–Pilot Study 1: (a) measurements of current dyspnea on admission and (b) measurements of exertional dyspnea prior to admission; Panel B–Pilot Study 2: (a) measurements of current dyspnea during first nursing shift and (b) measurements of any elevated dyspnea throughout the hospitalization. Error bars represent 95% confidence intervals calculated using Wald confidence limits for binomial proportions; tests of difference were performed using chi-squared tests with the exception of Pilot Study 2, upper right panel, which used Fisher’s exact test given small cell sizes.

Dyspnea prior to admission was significantly associated with adverse outcome. Patients reporting dyspnea during minimal ADL (eMRC grades 5–7) had 2.5 times the odds of experiencing a serious adverse event as those reporting dyspnea only when walking uphill or strenuously exercising (17% vs. 8%, odds ratio 2.4, 95% CI 1.2–4.7, p = 0.02) (Fig 3).

*Post-hoc* descriptive analysis showed that patients with higher exertional dyspnea also had significantly longer average hospital stays (4.4 days versus 2.9 days, p = 0.01) and tended to have much longer ICU stays (24 days per 100 admissions versus 7 days per 100 admissions, not statistically significant p = 0.26). There was little difference in average total charges ($22,298 versus $22,572, p = 0.94) or risk of readmission at 30 days (13% versus 10%, p = 0.50).

The 13% of patients with current dyspnea ≥4 (at rest) at the time of Initial Patient Assessment tended to be at increased risk of experiencing a serious adverse event (17% vs. 11%, odds ratio 1.73; 95% CI 0.89–3.35, not statistically significant in this pilot sample p = 0.1) (Fig 3).

Patients with current dyspnea ≥4 at the time of initial patient assessment trended towards worse outcomes by several other measures analyzed *post-hoc*, but these differences were not significant. They had longer average length of stay (4.5 days versus 3.3 days, p = 0.14) and longer average intensive care unit length of stay (39 days per 100 admissions versus 16 days per 100 admissions, p = 0.33). There was little meaningful difference in average total charges ($25,983 versus $23,160, p = 0.59) and likelihood of readmission at 30 days (13% versus 13%, p = 1.0).

#### Study 2—Outcomes associated with Dyspnea Each Nursing Shift

Of the 367 inpatients in Study 2, 4 (1%) died, 35 (10%) were transferred to the ICU, and 44 (12%) required the assistance of the medical emergency response team (Table 1).

The 12 patients in Study 2 who rated current dyspnea ≥4 during the first shift had about 60% more adverse outcomes, about the same fractional increase as patients who rated current dyspnea ≥4 at initial assessment in Study 1; however, the correlation in the smaller Study 2 sample was not significant (odds ratio 1.58, 95% CI 0.41–5.99, p = 0.50).

Serious adverse events during hospitalization were strongly associated with dyspnea rating ≥ 4/10 at any time during the stay rose (odds ratio 2.85, 95% CI 1.51–5.37, p = 0.0009). The time resolution of the study design did not allow us to reliably determine whether the increase in dyspnea preceded the adverse event in many cases.

## Discussion

### Dyspnea Prevalence

A large percentage of Study 1 patients reported significant dyspnea related to exertion prior to admission, and 13% reported active, burdensome dyspnea at the time of initial nursing assessment on admission. We found that 16% of the Study 2 inpatient population experienced dyspnea above the benchmark at some time during their hospital stay. In many of these patients the discomfort persisted for more than one nursing shift. Thus, burdensome dyspnea was nearly half as common as burdensome pain (Fig 1), and represents a substantial problem for symptom management.

### Association of dyspnea with Adverse Outcomes

Exertional dyspnea shortly before admission predicted adverse events in hospital in Study 1, and there was a trend toward more adverse events in patients with dyspnea at the time of admission to the unit. These findings from Study 1 suggest that dyspnea assessment may be a simple and economical way to improve risk prediction. The associations of outcome with dyspnea in Study 2 cannot, however, be interpreted as predictions because the temporal relationship between dyspnea and adverse event is not clear. In a number of cases, rapid response team activation preceded the first routine documented increase in dyspnea; because nurses were not required to document the time of onset of dyspnea, the actual time relationship is not known. This study utilizes data at the outset of systematic dyspnea documentation in our hospital; as familiarity and awareness increase nurses may exercise the option of recording dyspnea at any time to document time of dyspnea onset.

### Critique

Our two studies have several weaknesses. First, these were pilot studies designed to provide initial information on prevalence, and provide information on which to base further studies of dyspnea and associated risk, as well as investigate the feasibility of routine dyspnea evaluation in hospital wards (units). Because the number of adverse outcomes is low, these pilot studies are only powered to detect very strong associations of dyspnea with negative outcomes. Although both studies involved hundreds of patients, there were only a few dozen adverse events; based on these results, we project that a study would require approximately 1,000 patients to be powered to meaningfully detect a difference using binary categories, and correspondingly more to define a continuous relationship and to account for confounding factors. Second, one of the *a priori* adverse outcomes, summoning the rapid response team, may not be entirely independent of the dyspnea education effort that accompanied institution of routine measurement, as increased awareness of dyspnea may contribute to the nurse’s decision to summon the team (a good thing for patients, but one that confounds interpretation). Third, dichotomizing dyspnea at ≥4 for risk assessment is somewhat arbitrary, and larger data sets would allow dyspnea to be treated as a continuous variable or to investigate temporal patterns of dyspnea as predictors of adverse events.

Our study also has several strengths. First, this is the first prevalence information of its kind and provides new information about a common but aversive symptom for hospitalized patients. Second, despite the modest size of the individual study populations, the suggestion that dyspnea is a strong risk predictor is strengthened by the fact that in these 2 independent cohorts dyspnea during the first shift was associated with nearly the same odds of poor outcome (1.58 and 1.73). Pooling of the two studies, adding the 12 patients reporting dyspnea now ≥4 during the first shift in Study 2 to the 77 patients reporting dyspnea now ≥4 in the initial assessment in Study 1, with a binary covariate for study assignment, did not result in statistically significant confidence intervals (OR 1.90, 95% CI 0.94–3.07). Dyspnea during the admission shift is a ‘snapshot’ of patients with differing admission and treatment histories–some have been in the ED undergoing treatment for many hours, some have just arrived. Third, nursing adherence rate (percent of required assessments documented) for the Initial Patient Assessment conducted by nurses in the first pilot was 63%, and in the second longitudinal project, was 86% (nurses recorded an average of 1.86 dyspnea ratings each day; the average shift length in these units is 11.4 hours, thus 2.15 shifts per day). Similar studies of adherence to pain documentation show adherence rates of 63–83%.[32–35] The difference in adherence rates between our 2 studies likely reflects the challenges with integrating dyspnea assessment into nursing workflow using different methods.

## Conclusion

While our two pilot studies are modest in scope, this is the first report of routine quantitative dyspnea assessment and associated risk in general hospitalized patients. We found that a significant number of patients experience burdensome dyspnea during their hospital stay. We also found that nurses reliably performed routine dyspnea assessments on inpatients. We found good evidence that dyspnea assessment may be a useful tool to evaluate risk of adverse event. Routine documentation of patients’ ratings of dyspnea has the potential to drive improved symptom management interventions, better care, and better resource allocation.
